# Comparison Between Treatment Strategies of Carotid Stenosis in Patients Undergoing Coronary Artery Bypass Grafting

**DOI:** 10.21470/1678-9741-2020-0425

**Published:** 2022

**Authors:** Fernando Bassan, Vitor M P Azevedo, Ana Angélica Alves Pimenta Santos, Renan Bernardes de Mello, Annelise de Almeida Verdolin, Roberto Bassan

**Affiliations:** 1 Coronary Artery Disease Department, Instituto Nacional de Cardiologia, Rio de Janeiro, Rio de Janeiro, Brazil.; 2 Education & Research Department, Instituto Nacional de Cardiologia, Rio de Janeiro, Rio de Janeiro, Brazil.; 3 Cardiology Department, Pontifícia Universidade Católica do Rio de Janeiro, Rio de Janeiro, Rio de Janeiro, Brazil.

**Keywords:** Carotid Stenosis, Coronary Artery Bypass/surgery, Stroke, Carotid Endarterectomy, Coronary Artery Disease, Morbidity

## Abstract

**Introduction:**

In patients undergoing coronary artery bypass grafting (CABG), stroke is a major complication that increases morbidity and mortality. The presence of carotid stenosis (CS) increases risk of stroke, and the optimal treatment remains uncertain due to the lack of randomized clinical trials. The aim of this study is to compare three management approaches to CS in patients submitted to CABG.

**Methods:**

From 2005 to 2015, 79 consecutive patients with significant CS submitted to CABG were retrospectively evaluated. Patients were divided in three groups, according to CS treatment: 17 underwent staged carotid endarterectomy (CEA)-CABG, 26 underwent synchronous CEA-CABG, and 36 underwent isolated CABG without carotid intervention. The primary outcomes were composed by 30-day postoperative acute myocardial infarction (MI), 30-day postoperative stroke, and death due to all causes during the follow-up.

**Results:**

Patients were evaluated during an average 2.05 years (95% confidence interval = 1.51-2.60) of follow-up. Major adverse cardiac events, including death, postoperative MI, and postoperative stroke, occurred in 76.5% of the staged group, 34.6% of the synchronous group, and 33.3% of the isolated CABG group (*P*=0.007). As for MI, the rates were 29.4%, 3.85%, and 11.1% (*P*=0.045), respectively. There was no statistically significant difference in total mortality rates (35.3%, 30.8%, and 25.0%, respectively; *P*=0,72) and stroke (29.4%, 7.7%, and 8.3%, respectively; *P*=0,064) between groups.

**Conclusion:**

Staged CEA-CABG is associated with higher major adverse cardiac events and MI rate when compared to the strategy of synchronous and isolated CABG, but without statistically difference in total mortality during the entire follow-up.

**Table:** 

Abbreviations, acronyms & symbols	
AF	= Atrial fibrillation
CABG	= Coronary artery bypass grafting
CEA	= Carotid endarterectomy
ClCr	= Creatinine clearance
CPB	= Cardiopulmonary bypass
CS	= Carotid stenosis
EuroSCORE	= European System for Cardiac Operative Risk Evaluation
LAD	= Left anterior descending artery
LMCA	= Left main coronary artery
MI	= Myocardial infarction
NSTEMI	= Non-ST-elevation myocardial infarction
PAD	= Peripheral artery disease
STEMI	= ST-elevation myocardial infarction
TIA	= Transient ischemic attack

## INTRODUCTION

Among patients with coronary artery disease, simultaneous carotid obstruction may occur in up to 22% of the cases^[1,2]^. Perioperative stroke is a severe coronary artery bypass grafting (CABG) complication with an incidence ranging from 0.8 to 5.2%^[3,4]^, being strongly related to the degree of the carotid stenosis (CS)^[5-7]^ and tripling cardiac surgery mortality^[8]^. Several factors contribute to its occurrence such as age, diabetes mellitus, smoking, aortic calcification, atrial fibrillation (AF), cardiopulmonary bypass (on-pump time), and pre-existence of carotid artery disease^[9-13]^.

Also, in 60% of these patients there is no relationship between the ischemia site and the carotid obstruction territory, and 76% of strokes occur in patients without significant CS^[14,15]^.

In the lack of randomized clinical trials, there is no conclusive data to establish the best approach to patients with CS undergoing CABG^[16-21]^.

In this study, we present the results of the Instituto Nacional de Cardiologia (or INC), a fourth level hospital and reference of the Brazilian Health Ministry to cardiac surgery, for the management of patients with concomitant CS referred to CABG.

## METHODS

### Study Population

In a retrospective, observational, and cohort type study, consecutive, non-selected patients with CS — defined as ≥ 70% unilateral or ≥ 50 bilateral obstruction, with or without prior neurological symptoms — were evaluated by carotid artery ultrasound and submitted to CABG, from January 2005 to December 2015. Patients with concurrent indication for valve or aortic repair or previously submitted to cardiac surgery have been excluded from this study.

### Ethics Approval and Consent to Participate

This study was approved by the hospital’s Ethics Committee (23615413.0.0000.5272). All the procedures in this study were in accordance with the 1975 Helsinki Declaration, updated in 2013. Informed consent was obtained from all participants included in the study.

### Patients’ Management

According to the institutional protocol, the search for CS during the preoperative period is routinely performed with carotid artery ultrasound in patients aged ≥ 65 years, as well as for the presence of left main coronary artery disease, peripheral artery disease (PAD), carotid bruit on physical examination, or prior history of neurologic events such as stroke or transient ischemic attack. When a considerable CS is detected, all patients are submitted to further carotid arteriography for confirmation and better analysis. CS management was decided at the discretion of a multidisciplinary team composed of clinical cardiologists, neurologist, and cardiac and vascular surgeons, in three possible ways: staged, when carotid endarterectomy (CEA) was performed in the same hospitalization, however prior to CABG; synchronous, when CEA and CABG were performed at the same surgical time; and isolated, when CABG was performed without carotid intervention. Carotid interventions were performed by vascular surgeons and coronary revascularizations by cardiac surgeons. The European System for Cardiac Operative Risk Evaluation (EuroSCORE) I was used to assess the preoperative risk.

### End Point

The primary outcome was defined by the occurrence of postoperative stroke, postoperative myocardial infarction (MI), and death due to all cause during the entire follow-up period. Postoperative stroke was defined as a new or worsening focal neurological event that persisted for > 24 hours during the hospitalization period. Postoperative MI was defined by an elevation of cardiac biomarkers (creatine kinase-myocardial band or troponin) > 5 times the 99^th^ percentile upper reference limit plus either new pathological Q waves in the electrocardiogram, or imaging evidence of a new loss of viable myocardium, or angiographically documented new graft or native coronary artery occlusion during the hospitalization period.

### Data Collection

The computerized database of the Coronary Artery Department was queried to identify all patients included on this trial. Preoperative, intraoperative, and postoperative clinical data were obtained through hospital chart review and telephone calls were made to collect vital status at the end of the follow-up.

### Statistical Analysis

**Fig. 1:**
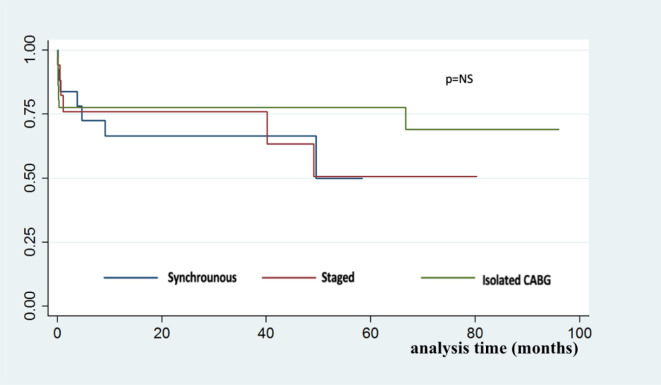
Discloses the Kaplan-Meier survival curves during the median of 2.05 years of follow-up according to surgical treatments. CABG=coronary artery bypass grafting

Statistical analyses were performed using the Stata program software v. 14.2 (StataCorp LLC). The differences between groups were evaluated using the one-way analysis of variance test for continuous variables and the Fisher’s exact test and Chi-squared test for binary category variables. Long-term outcomes data were analyzed using the Kaplan-Meier method (Figure 1) and log-rank test. Logistic regression was used for univariate and multivariate analysis. All *P*-values were two sided, with *P*<0.05 considered as significant.

## RESULTS

### Patient Recruitment and Baseline Characteristics

**Fig. 2:**
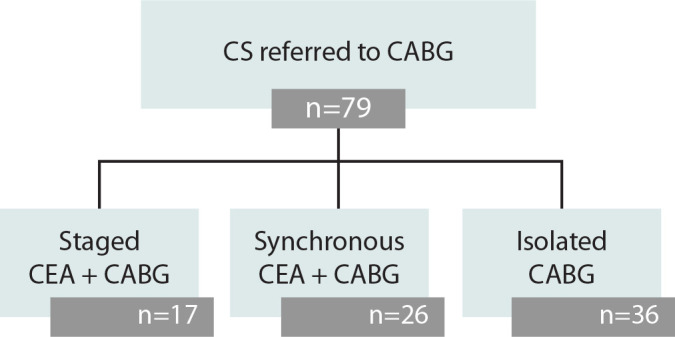
Enrollment and treatment assignment. CABG=coronary artery bypass grafting; CEA=carotid endarterectomy; CS=carotid stenosis

During the pre-specified period, 79 patients with relevant CS and submitted to CABG have been followed by an average period of 2.05 years (95% confidence interval = 1,51-2,60). Of these patients, 17 underwent staged CEA-CABG, 26 underwent synchronous CEA-CABG, and 36 underwent isolated CABG (Figure 2).

There were no significant differences in preoperative clinical variables among the three groups regarding age, gender, diabetes mellitus, on-pump time, off-pump CABG, creatinine clearance, and ejection fraction, except for the higher rate of previous AF on the staged group. Also, carotid and coronary angiographic characteristics were not statistically different among the assigned groups (Table 1).

**Table 1:** Patients’ demographic and baseline characteristics.

	Staged CEA-CABG (n=17)	Synchronous CEA-CABG (n=26)	Isolated CABG (n=36)	
Age (years)	68.2±7.03	66.2±5.64	66.5±7.53	*P*=0.247
Male gender, % (n)	70.6 (12)	57.7 (15)	61.1 (22)	*P*=0.688
Diabetes, % (n)	58.8 (10)	46.1 (12)	50 (18)	*P*=0.715
PAD, % (n)	47.1 (8)	69.2 (18)	38.9 (14)	*P*=0.059
Previous AF, % (n)	17.6 (3)	0 (0)	2.8 (1)	*P*=0.040
Previous TIA/stroke, %	41.2	23.1	55.6	*P*=0.038
Mean CPB time (min)	58.3	77.3	61.9	*P*=0.272
Off-pump CABG, %	19.2	29.4	13.9	*P*=0.404
EuroSCORE I, %	5.2±2.6	8.2±5.3	5.3±2.7	*P*=0.012
Ejection fraction	61.4±11.7	58.7±16.0	62.4±13.5	*P*=0.549
ClCr (ml/min)	67.3±25.7	68.9±23.1	66.9±23.2	*P*=0.922
**Preoperative diagnosis**				
STEMI, % (n)	5.9 (1)	3.8 (1)	0 (0)	*P*=0.389
NSTEMI, % (n)	5.9 (1)	15.4 (4)	11.1 (4)	*P*=0.630
Unstable angina, % (n)	17.6 (3)	23.1 (6)	13.9 (5)	*P*=0.646
Stable angina, % (n)	64.7 (11)	46.1 (12)	72.2 (26)	*P*=0.110
Carotid artery disease				
Unilateral CS ≥ 70%, % (n)	35.3 (6)	50 (13)	33.2 (12)	*P*=0.387
Bilateral CS ≥ 70%, % (n)	35.3 (6)	19.2 (5)	22.2 (8)	*P*=0.455
**Coronary artery disease**				
LMCA > 50%, % (n)	41.2 (7)	53.9 (14)	44.4 (16)	*P*=0.665
Proximal LAD, % (n)	52.9 (9)	57.7 (15)	77.8 (28)	*P*=0.116
3-vessel disease, % (n)	82.4 (14)	80.8 (21)	75 (27)	*P*=0.961

AF=atrial fibrillation; CABG=coronary artery bypass grafting; CEA=carotid endarterectomy; ClCr=creatinine clearance; CPB=cardiopulmonary bypass; CS=carotid stenosis; EuroSCORE=European System for Cardiac Operative Risk Evaluation; LAD=left anterior descending artery; LMCA=Left main coronary artery; NSTEMI=non-ST-elevation myocardial infarction; PAD=peripheral artery disease; STEMI=ST-elevation myocardial infarction; TIA=transient ischemic attack

The synchronous group presented a higher EuroSCORE I risk when compared to the staged and isolated surgery groups. The isolated group had higher rate of prior cerebrovascular events, followed by the synchronous and staged surgery groups. Despite not being statistically significant, PAD was most common in the synchronous group, followed by the staged and isolated groups.

### End Points

During the follow-up, the primary outcome occurred in 13 of the 17 patients assigned for the staged group, nine of 26 patients assigned for synchronous surgery, and 12 of 36 patients for isolated CABG (Table 2).

**Table 2:** Clinical outcomes.

	Staged CEA-CABG (n=17)	Synchronous CEA-CABG (n=26)	Isolated CABG (n=36)	
Primary outcome	76.5% (13)	34.6% (9)	33.3% (12)	*P*=0.007
Death	35.3% (6)	30.8% (8)	25% (9)	*P*=0.725
MI	29.4% (5)	3.85% (1)	11.1% (4)	*P*=0.045
Stroke	29.4% (5)	7.7% (2)	8.3% (3)	*P*=0.064
Postoperative AF	23.5% (4)	23.1% (6)	27.8% (10)	*P*=0.899

AF=atrial fibrillation; CABG=coronary artery bypass grafting; CEA=carotid endarterectomy; MI=myocardial infarction

In an individual outcome analysis, MI incidence was significantly higher in the staged group, followed by the isolated and the synchronous surgery groups. Although not statistically significant, stroke incidence was far more frequent in the staged group in comparison with the isolated and synchronous CABG groups. It is important to notice that the staged group also presented higher prevalence of previous AF. All-cause mortality did not differ between the staged, isolated, and synchronous groups during the follow-up, as well as the rate of postoperative AF (Table 2).

## DISCUSSION

Our study showed that staged carotid and cardiac surgery presents a higher rate of composed outcomes, mainly at the expense of MI, when compared to the synchronous and isolated groups, but no significant difference was seen in all-cause mortality between all groups during the 2.05 years of follow-up.

In a retrospective and non-randomized study, Shishebor et al.^[17]^ showed that the compound outcome of death, MI, and stroke was higher in the staged group, at the expense of an MI rate 50 times higher when compared to the synchronous strategy (24% *vs*. 0.51%; *P*<0.001), but without one-year mortality difference, a similar result to ours.

Another observational databased study by Gopaldas et al.^[22]^ with more than 22,000 patients presented similar results, with a higher cardiovascular complication rate (odds ratio 1.51, *P*<0.001) in the staged *vs*. synchronous group, but without mortality difference. An interesting analysis is that cardiac complications were higher when the staged approach was initiated by CEA (15.1% *vs*. 10.4%, *P*<0.001), whereas neurological complications were more pronounced when CABG was made first (7.1% *vs*. 3.1%, *P*<0.001).

But conflicting results exist. A meta-analysis by Chan et al.^[23]^ involving only observational studies and developed on the last three decades showed that the staged group performed better with fewer strokes, when compared to synchronous CABG-CEA (2.8% *vs*. 3.6%, *P*<0.001). In accordance with the present study, there was no difference on mortality after an one-year follow-up (2.22% *vs*. 12.0%, *P*=0.33).

As most of the trials are observational and non-randomized, single-center and with relatively small sample size, the heterogeneity of the results may in part be explained by local surgical expertise and patient selection, as both CABG and CEA are highly complex procedures.

Usually, indications for CABG apply to patients with high-risk clinical findings such as severe coronary artery stenosis, moderate to severe ischemia, and/or uncontrolled angina symptoms. Thus, preoperative assessment will categorize those patients on such a high-risk profile that they wouldn’t be recommended to be submitted to any other interventions rather than myocardial revascularization. As CEA is considered a procedure of intermediate to high risk, it naturally exposes those patients to an elevated perioperative MI risk, justifying the results found on the staged group.

The interstaged period between CEA and CABG is challenging, as MI risk can affect 18.7% of those patients^[24]^. Using the synchronous approach, simultaneous revascularization may provide an ischemic protection effect for both coronary and carotid beds^[17]^.

Finally, two crucial points of convergence between the current and other studies are important: first, patients with concomitant significant carotid and coronary artery disease have high atherosclerotic burden, reflected by the expressive rate of cardiac and neurological complications during follow-up. Second, irrespective of this high number of complications and the handling strategy adopted, the mortality rate among groups does not differ.

### Limitations

This is a non-randomized retrospective cohort study with a relatively small sample size. The treatment assignment was chosen at discretion of the heart team as disclosed in methods, therefore, some selection bias can occur.

## CONCLUSION

In our retrospective study, the strategy of staged CEA-CABG was associated with a higher rate of the primary outcomes in comparison with synchronous CEA-CABG and isolated CABG, at the expense of MI and with a tendency, although non-significant, of a higher incidence of stroke during the follow-up of 2.05 years. Mortality rates due to all causes did not differ between groups. These results are similar to most of the main studies already published, but due to a complete lack of robust randomized clinical trial, the best approach is still unclear and should take into consideration an analysis between myocardial *vs*. cerebral risk.

**Table:** 

Authors' roles & responsibilities	
FB	Substantial contributions to the conception and design of the work; and the acquisition, analysis and interpretation of data for the work; revising the work critically for important intellectual content; final approval of the version to be published
VMPA	Substantial contributions to the analysis and interpretation of data for the work; revising the work critically for important intellectual content; final approval of the version to be published
AAAPS	Substantial contributions to the acquisition and analysis and interpretation of data for the work; revising the work critically for intellectual important content; final approval of the version to be published
RBM	Substantial contributions to the acquisition of data for the work; final approval of the version to be published
AAV	Substantial contributions to the acquisition of data for the work; final approval of the version to be published
RB	Substantial contributions to the analysis of data for the work; revising the work critically for important intellectual content; final approval of the version to be published
