# ERRATUM

**DOI:** 10.21470/1678-9741-2020-0425e

**Published:** 2022

In the article titled “Comparison Between Treatment Strategies of Carotid Stenosis in Patients Undergoing Coronary Artery Bypass Grafting”, DOI 10.21470/1678-9741-2020-0425, published in the Brazilian Journal of Cardiovascular Surgery, issue 37.3, the corresponding author’s e-mail address is incorrect.

In the original version, the information is:

E-mail: fbassan@globo.com

The correct information is:

E-mail: drfernandobassan@gmail.com

